# Supplementary material for: Effectiveness of small road tunnels and fences in reducing amphibian roadkill and barrier effects at retrofitted roads in Sweden
Source: PeerJ. 2019 Aug 26;7:e7518. doi: 10.7717/peerj.7518 (PMC6714967; doi:10.7717/peerj.7518)
Supplement: Supplemental Information 1 [file peerj-07-7518-s001.pdf]

Manuscript title

Effectiveness of small road tunnels and fences in reducing amphibian roadkill and barrier effects at retrofitted roads in Sweden

Jan Olof Helldin[1] and Silviu O. Petrovan[2,3]*

1) *Swedish Biodiversity Centre, Swedish University of Agricultural Sciences, Uppsala, Sweden*
2) *Department of Zoology, University of Cambridge, Cambridge, UK*
3) *Froglife, Peterborough, UK*

Supplemental article 1

Site descriptions

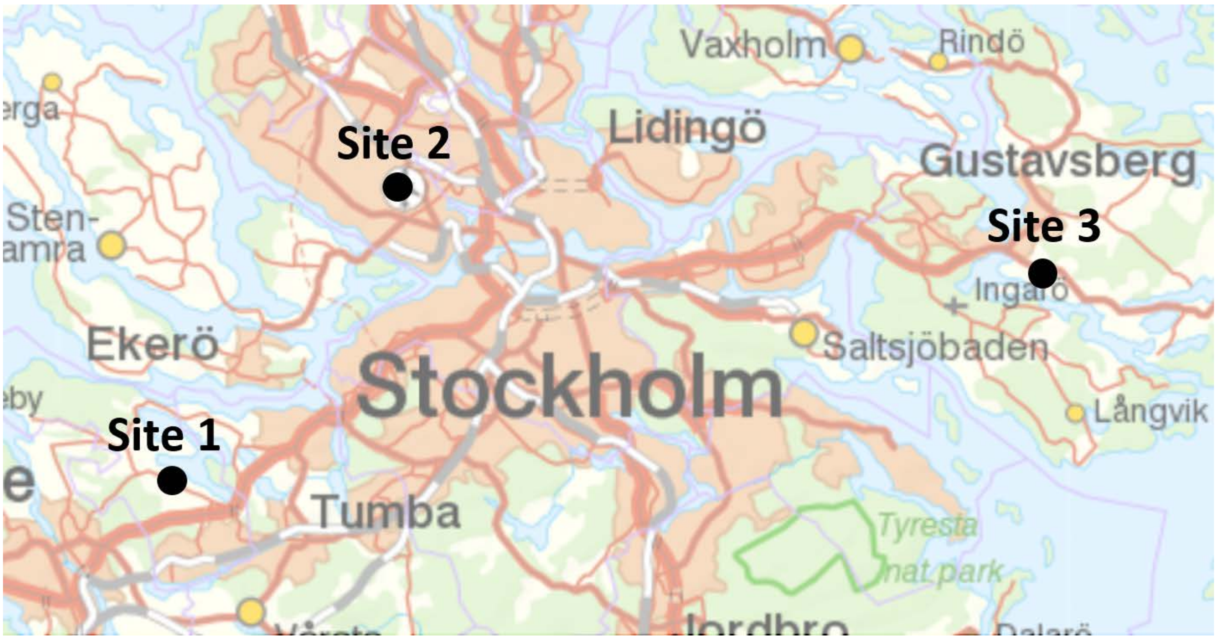

Figure S1. Overview of amphibian mitigation sites included in the study. Map image credit: Lantmäteriet.

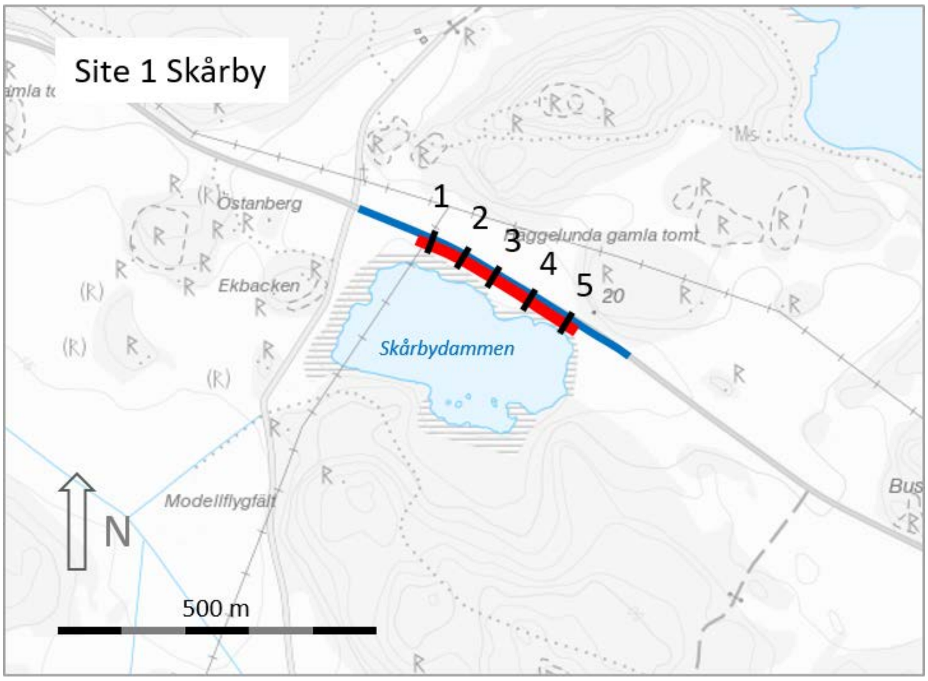

Figure S2. Map of Skårby and the wetland Skårbydammen. Red line denote mitigated (fenced) section, black lines are the tunnels, and blue line is the road section where amphibians were counted before and after mitigation. Map image credit: Lantmäteriet.

Site 1 Skårby

Site characteristics

The pond and wetland at Skårby (ca 6 ha) has one of Swedens largest breeding populations of great crested newt (>300 individuals) and also a large breeding population of smooth newt (>2000 individuals; Peterson & Collinder, 2006). The amphibian mitigation system at this site was constructed in phases; 300 m permanent fence with three tunnels was constructed in 2005 and two additional tunnels were constructed in 2008.

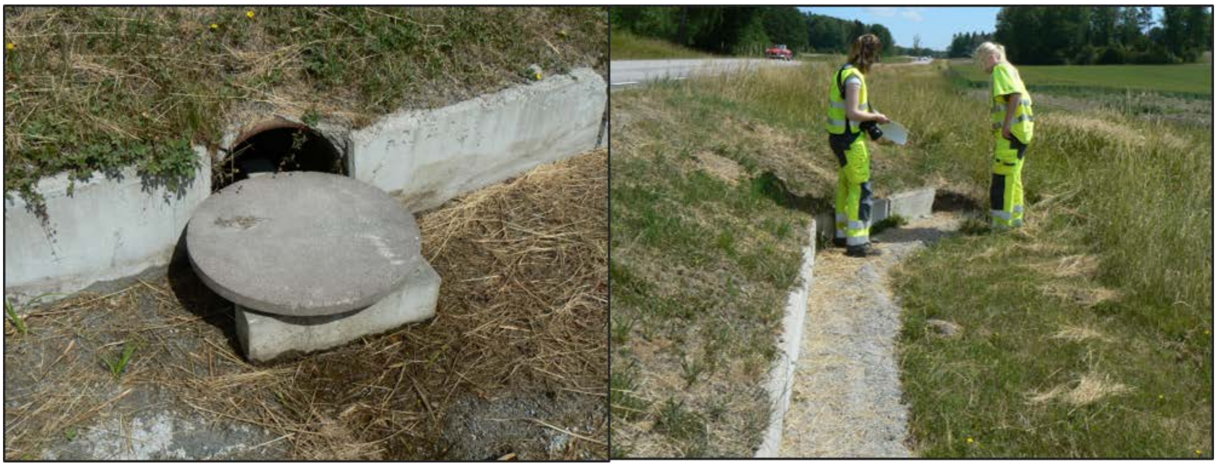

Figure S3. Amphibian tunnel with guiding structure, fence and fence-end at Skårby, Sweden. Photo: Jan Olof Helldin

Data collection

Before mitigation: Amphibians on the road were counted in the night 15–16 April 2004 (ca 10h). The night was selected to represent an important migration night (suitable weather conditions and timing). The road section searched was ca 520 m, extending in both directions ≥150 m outside of the section to be mitigated.

After mitigation: Amphibians on the road were counted during four mornings between 6 and 22 April 2008, selected to represent important migration nights. The road section searched was the same as before mitigation. Only fresh carcasses (accumulated during the previous night) were counted. Trapping in tunnels was conducted in two nights 9 and 11 April 2010, and in three nights 15–18 April 2013. Bow net traps were mounted on the tunnel exits (i.e., the opening on the wetland side) to count amphibians passing through the tunnels toward the wetland. Nets were mounted in the early evening and trapped amphibians were counted and released in the following morning. One of the tunnels (no. 2) could not be monitored because the exit was completely under water; however this tunnel was in place already before the mitigation system was constructed, functioning as a drainage pipe, and it was therefore was not further considered in the analyses.

Position, species, status (e.g., dead/alive) and time was recorded for all amphibian observations, both on the road and in the tunnels. Field methodology and data output from the site is described in further detail in technical reports (Ekologigruppen, 2004; Syde, 2008; Collinder, 2010; Peterson, 2013a). Amphibian counts along the road were conducted also in other years between 2004 and 2013 (see e.g. Peterson, 2013a) but we considered only the selected data as detailed and standardized enough to allow for a comparison between road sections and time periods.

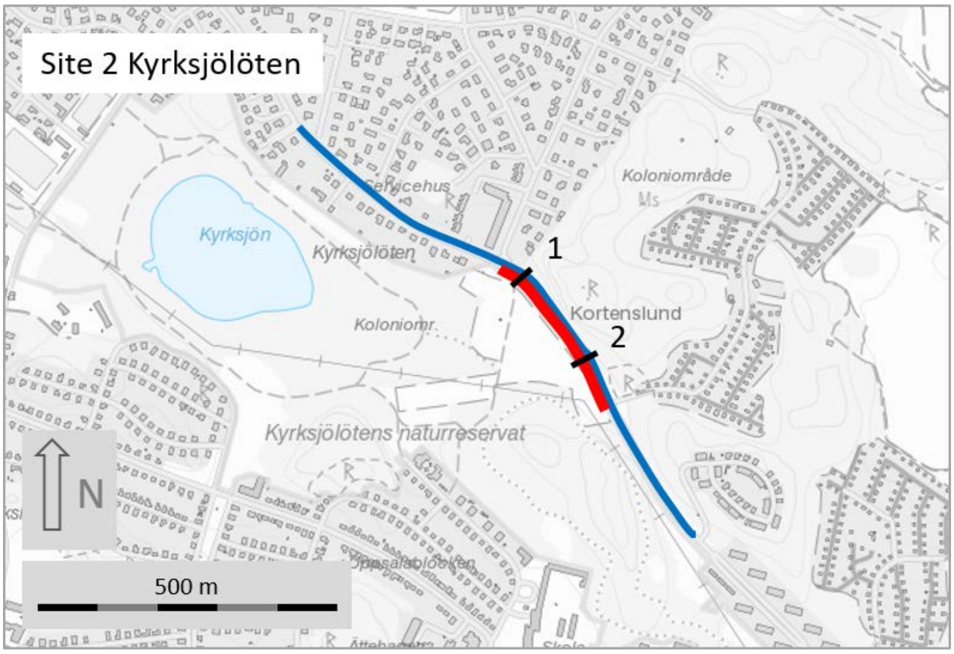

Figure S4. Map of Kyrksjölöten and lake Kyrksjön. Red line denote mitigated (fenced) section, black lines are the tunnels, and blue line is the road section where amphibians were counted before and after mitigation. Map image credit: Lantmäteriet.

Site 2 Kyrksjölöten

Site characteristics

The lake Kyrksjön (ca 5 ha) and adjacent wetlands in the nature reserve Kyrksjölöten has a large breeding populations of common toad (the exact number has however not been assessed). The numbers of other amphibians are small; great crested newt has recently been reintroduced. The toad population appears to have withstood housing and traffic development in the surrounding areas starting in 1940s, but have started declining in the last decades (Calluna, 2012). The amphibian mitigation system was constructed at the major road (Spångavägen) going past the area, in connection to an upgrade of the road in autumn 2014.

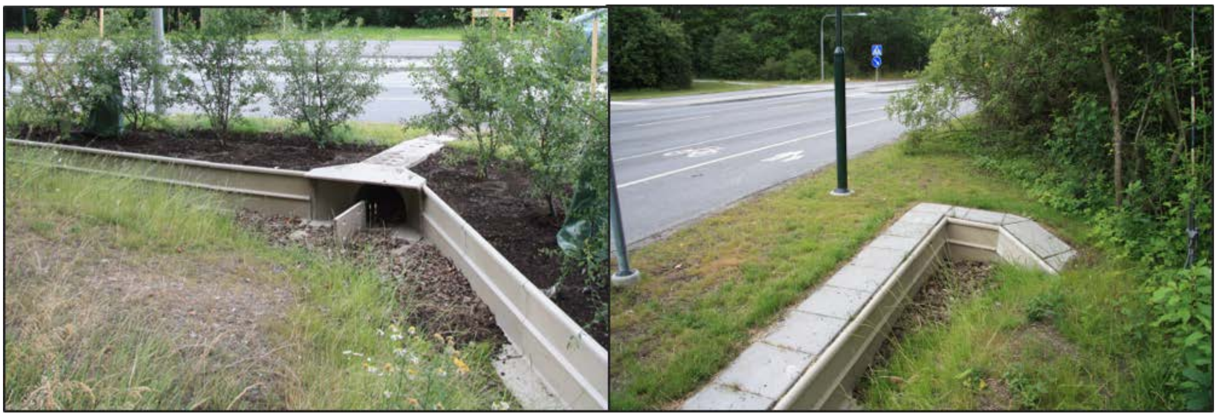

Figure S5. Amphibian tunnel with guiding structure, fence and fence-end at Kyrksjölöten, Sweden. Photo: Jan Olof Helldin and Erik Jondelius

Data collection

Before mitigation: Amphibians were counted in 17 evenings (during ca 3h starting at sunset) between 27 March and 9 May 2012, at a one-sided temporary fence along the section to be permanently mitigated, and by a search on the road, including the verge on the northern side. The total road section searched was ca 1000 m, thus extending in both directions >200 m outside of temporary fenced section. Pitfall traps along the fence were set each of these 17 evenings and emptied in the following morning. Amphibians found alive were moved to the wetland side of the road. Only in 7 of the 17 nights a significant number of amphibians were found or trapped, and accordingly could be labelled significant migration night.

After mitigation: Amphibians were counted in three evenings (during ca 3h starting at sunset) between 8 and 15 April 2015, along the permanent fences and on the road and verge, same road section as before mitigation. Evenings for fieldwork were selected to represent important migration evenings (suitable weather conditions and timing). The few live amphibians found on the road or in the verge were moved to the wetland side of the road, while animals along the fences were counted but left in place. Dead amphibians had accumulated between evenings, thus representing a total period of ca 8 days. Customized infrared time-lapse cameras (15s interval) assembled by Froglife (see Jarvis, Hartup & Petrovan, 2019) were mounted in the ceiling inside both tunnel entrances from 1 April to 3 May 2015; a total of 32 camera days. Only in 14 of the 32 days a significant number of amphibians were found or trapped, and accordingly could be labelled significant migration night.

Position, species, status (e.g., dead/alive) and time was recorded for all amphibian observations, both on the road, along fences and in the tunnels. For animals on tunnel photos, movement direction (in or out) was noted and the net number through the tunnels was calculated. Field methodology and data output from the site is described in further detail in technical reports (Calluna, 2012; Helldin, 2015).

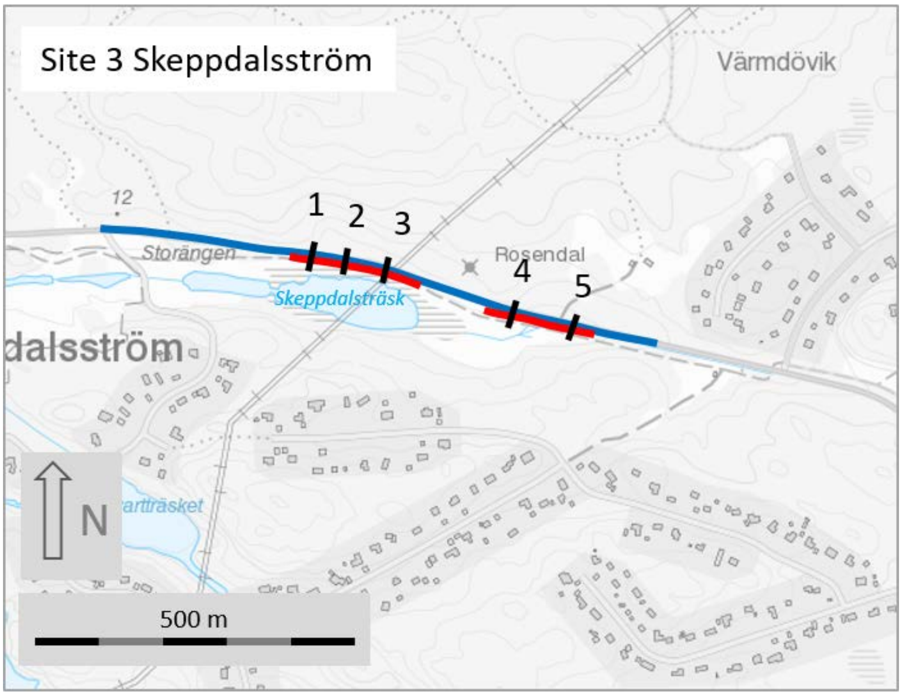

Figure S6. Map of Skeppdalsström and the wetland Skeppdalsträsk. Red line denote mitigated (fenced) section, black lines are the tunnels, and blue line is the road section where amphibians were counted before and after mitigation. Map image credit: Lantmäteriet.

Site 3 Skeppdalsström

Site characteristics

The wetland Skeppdalsträsk (ca 4 ha) serves as breeding area for all five amphibian species. Breeding populations during studies were estimated to 600 common toads, 150 common frogs and 60 moorfrogs (Andersson & Lundberg, 2015); smooth newt was not included in the assessment but should be in level with common toad, while great crested newt was not discovered until 2017 (Anne Crussell, pers. comm.). Volunteers have been active on the site since 2013 to move amphibians across the road during spring migration. The amphibian mitigation system was constructed in summer 2015.

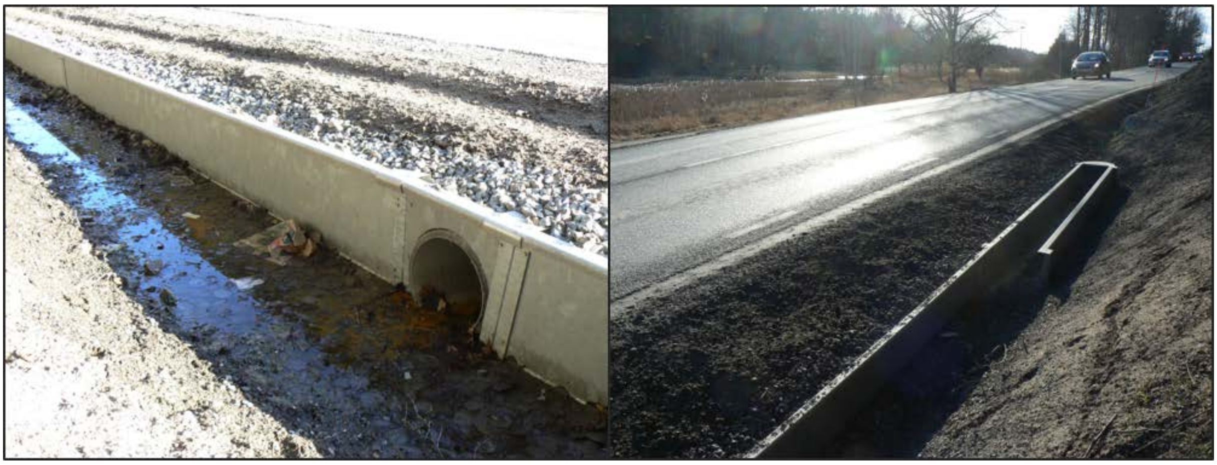

Figure S7. Amphibian tunnel, fence and fence-end at Skeppdalsström, Sweden. Photo: Jan Olof Helldin

Data collection

Before mitigation: Amphibians were counted in seven evenings (during ca 3h starting at sunset) between 7 and 19 April 2015, by a search on the road, including the verge on the northern side. Evenings for fieldwork were selected to represent important migration evenings (suitable weather conditions and timing). Each evening, at least 5 persons took part in the search, regularly patrolling the road, and accordingly most amphibians were found alive before or when entering the road. The road section searched was ca 950 m, thus extending between and in both directions ≥100 m outside of the mitigated sections. All amphibians found alive were moved to the wetland side of the road.

After mitigation: Amphibians were counted in four evenings (during ca 3h starting at sunset) between 7 and 18 April 2016, along the permanent fences and on the road and verge, same road section as before mitigation. Evenings for fieldwork were selected to represent important migration evenings (suitable weather conditions and timing). Similar to before mitigation, at least 5 persons took part in the search each evening, patrolling the unmitigated part of the road, and accordingly most amphibians were found before getting killed. Live amphibians found on the road or in the verge were moved to the wetland side of the road, while animals along the fences were left in place. Customized infrared time-lapse cameras (15s interval) assembled by Froglife (see Jarvis, Hartup & Petrovan, 2019) were mounted in the ceiling inside two of the tunnel entrances 5–13 April (tunnels no. 3–4) and two of the other tunnel entrances 13–23 April 2016 (tunnels no. 1–2). Due to temporary failure of the IR light source, the total number of camera days acquired varied between 7 and 11. One of the tunnels (no. 5) was not monitored because of a constant flow of water inside the tunnel, which was assumed to interfere with the analysis of tunnel photos; however this tunnel was in place already before the mitigation system was constructed, functioning as a drainage pipe, and it was therefore was not further considered in the analyses.

Position, species, status (e.g., dead/alive) and time was recorded for all amphibian observations, both on the road, along fences and in the tunnels. For animals on tunnel photos, movement direction (in or out) was noted and the net number through the tunnel was calculated. Field methodology and data output from the site is described in further detail in technical reports (Peterson, 2013b; Andersson & Lundberg, 2015; Helldin, Olsson & Andersson, 2018).

References

Andersson P, Lundberg J. 2015. Groddjursinventering och flytt vid väg 222, Skeppdalsström, Värmdö kommun, Stockholms län. *Report from Trafikverket* dated 2015-06-10. (In Swedish) http://media.triekol.se/2019/03/Andersson-Lundberg-2015-Groddjursinventering-och-flytt-vid-vag-222.pdf

Calluna. 2012. Groddjursinventering; Undersökning av vandringsmönster inför anläggning av groddjurstunnlar utmed Spångavägen vid Kyrksjölötens naturreservat. *Report from Calluna AB and Trafikkontoret, Stockholms Stad*. (In Swedish) http://miljobarometern.stockholm.se/content/docs/tema/natur/Groddjur/Groddjursrapport_Spangavagen121219_Lagupplost.pdf

Collinder P. 2010. Groddjursinventering 2010 vid konfliktpunkter i Stockholms län. *Report from Ekologigruppen AB* August 2010. (In Swedish) http://media.triekol.se/2019/03/Collinder-2010-Groddjursinventering-2010-vid-konfliktpunkter-i-Stockholms-lan.pdf

Ekologigruppen. 2004. Utredning av behov av groddjurspassage vid Skårbydammen. *Report from Ekologigruppen AB* 2004-05-06. (In Swedish) http://media.triekol.se/2019/03/Ekologigruppen-2004-Utredning-Skarby.pdf

Helldin JO. 2015. Uppföljning och utvärdering av groddjurstunnlar på Spångavägen. *Report from Calluna AB and Stockholm Stad*. (In Swedish) http://miljobarometern.stockholm.se/content/docs/tema/natur/Groddjur/Groddjurstunnlar%20Spångavägen%20Rapport%202015-10-02.pdf

Helldin JO, Olsson T, Andersson P. 2018. Uppföljning och utvärdering av groddjursåtgärder vid Skeppdalsström – G178, Värmdö kommun. *Trafikverket report* 2018:232. (In Swedish) https://trafikverket.ineko.se/Files/sv-SE/57352/Ineko.Product.RelatedFiles/2018_232_uppfoljning_och_utvardering_av_groddjursatgarder_vid_skeppdalsstrom.pdf

Jarvis LE, Hartup M, Petrovan SO. 2019. Road mitigation using tunnels and fences promotes site connectivity and population expansion for a protected amphibian. *European Journal of Wildlife Research* 65:27.

Peterson T. 2013a. Utvärdering av grodtunnlar vid Skårbydammen, Salem våren 2013. *Report from Trafikverket*, ärendenummer TRV 2013/5489. (In Swedish) http://media.triekol.se/2019/03/Peterson-2013-Utvardering-av-grodtunnlar-i-Skarbydammen.pdf

Peterson T. 2013b. Utredning av groddjursvandring över väg 222 vid Skeppdalsström. *Report from Trafikverket*, ärendenummer TRV 2013/5489. (In Swedish) http://media.triekol.se/2019/03/Peterson-2013-Utredning-inför-grodtunnlar-vid-Stavnasvagen.pdf

Peterson T, Collinder P. 2006. Utvärdering av grodtunnlar vid Skårbydammen, Salem våren 2006. *Report from Ekologigruppen AB*. (In Swedish) http://media.triekol.se/2019/03/Ekologigruppen-2006-Utvardering-av-grodtunnlar-vid-Skarbydammen.pdf

Syde N. 2008. Groddjursinventering vid konfliktpunkter i Stockholms län. *Report from Ekologigruppen AB*. (In Swedish) http://media.triekol.se/2019/03/Syde-2008-Groddjursinventering-vid-konfliktpunkter-i-Stockholms-lan.pdf
